# High resolution carbon stock and soil data for three salt marshes along the northeastern coast of North America

**DOI:** 10.1016/j.dib.2018.07.037

**Published:** 2018-07-21

**Authors:** Lee B. van Ardenne, Serge Jolicoeur, Dominique Bérubé, David Burdick, Gail L. Chmura

**Affiliations:** a Department of Geography, McGill University, Montreal, QC, Canada; b Département d’histoire et de géographie, Université de Moncton, Moncton, NB, Canada; c Department of Energy and Resource Development, Geological Surveys Branch, NB, Canada; d Department of Natural Resources & the Environment, Jackson Estuarine Laboratory, Durham, NH, USA

## Abstract

The data presented here includes a table of soils measurements taken at high resolution depth intervals (5 cm) for three salt marshes, two along the New Brunswick coast of Canada and one on the southern coast of Maine, USA. The data includes a table which includes the bulk density, percent organic matter, percent organic carbon, carbon stock, and rhizome dominance (if identifiable) at 5 cm depth intervals for each soil core. Shapefiles are also included which indicates the GPS position of acquired cores and sites where marsh depth was measured but no material was recovered. These shapefiles also include marsh peat depth and estimates of carbon stock for each point. For further information and interpretation of the included data please see the companion research article titled “The Importance of Geomorphic Context for Estimating the Carbon Stock of Salt Marshes” [1].

**Specifications Table**

**Table:** 

Subject area	*Geography*
More specific subject area	*Physical Geography*
Type of data	*Primary data*
How data was acquired	*Extraction and analysis of soil cores.*
Data format	*Raw*
Experimental factors	*Soil samples dried at 60* *°C until no mass lost between measurements (~ 12* *h typically). Loss on ignition (LOI) performed at 350* *°C for one hour followed by four hours at 550* *°C.*
Experimental features	*We measured bulk density, C density, and estimated carbon stocks of three marshes: Two formed in association with a developing sand spit along the Gulf of St. Lawrence coast of New Brunswick, Canada, and another with a lagoon on the coast of Maine, USA. Data represented here includes all soils data and GPS points or collection sites, with carbon stock estimates.*
Data source location	*Department of Geography, McGill University, Montreal, Canada.*
Data accessibility	*Data included in this article.*
Related research article	*Related to* Van Ardenne LB, Jolicoeur S, Bérubé D, Burdick D, Chmura GL. 2018. The Importance of Geomorphic Context for Estimating the Carbon Stock of Salt Marshes. *Geoderma* 330: 264–275 [1].

**Value of the data**

• Data records carbon density at a much higher resolution and to a greater depth than is usually recorded in salt marsh studies, which provides insight into changes and carbon storage with depth.
• The included data allows for the estimation of carbon stocks in salt marshes in a higher latitude that those studies used to develop the IPCC default estimation values [2], which if combined with further data from further regions and differing environmental conditions may allow for the development of more accurate regional estimates of salt marsh carbon storage.
• The data could be complied with a larger database of marsh samples with various geomorphic contexts to help derive typical carbon storage or accumulation conditions for specific marsh subtypes (fluvial, back-barrier, etc.).

## 1 Data

The data includes a table which includes the bulk density, percent organic matter, percent organic carbon, carbon stock, and percent rhizome dominance (where identifiable) for each 5 cm depth interval of soil cores recovered from three salt marshes on the Atlantic coast of New Brunswick, Canada, and Maine, USA (Table 1). The data table also indicates the corer used to collect each subsample, transect and flag names, and which marsh each was collected at (see specification table for marsh names and locations). Rhizome dominance is organized as one species per column and is expressed as the percentage of identifiable rhizomes in the subsample which are of the relevant species; they collectively sum to 100 for each subsection. Percent organic matter is expressed as a proportion (0–1) of the dry mass of each subsection. All other data in the table is represented in the units indicated in the column name. Corers used to collect the data include 25 and 60 mm gauge corers, indicated in the table as “25 mm” and “60 mm” respectively, and two similar Russian peat corers “T-Russian” and “G-Russian” respectively.

**Table 1:** Location of sample sites.

Marsh location/name	Latitude	Longitude
Wells Marsh, ME, USA	43°18′58″ N	70°34′15″ W
Grants Beach, NB, Canada	46°10′21″ N	64°3′32″ W
Point Carron, NB, Canada	47°39′03″ N	65°36′44″ W

The included shapefiles contain the GPS positions of acquired cores and sites where marsh depth was measured but no material was recovered. These shapefile attribute tables contain marsh peat depth and estimates of carbon stock for each point, and well as the relevant core name. Core names which do not have an analogue in the core data table are sites where only depth was measured. The shapefile for Wells has been spit into two sections, with one containing all the data points in the marsh interior while the other contains the points on a small back barrier section.

## 2 Experimental design, materials and methods

Full detail of the core collection and processing procedures are described in the associated research article [1]. Cores were collected along transects from the upland to the seaward edge of the marshes and vegetation was recorded within an ~ 0.5 m radius around each site where a core was collected, or depth recorded. Locations of each sample site was recorded using a Lecia Viva differential GPS.

Soil cores were extracted using a variety of hand corers, with additional soil depths determined using either an aluminum probe rod (Wells only) or via the use of the corers. Cores were packed, placed on ice in the field and stored at 4 °C once returned to the field base and lab. All sites were cored until refusal. 46 soil cores were collected, and 157 further soil depths were recorded.

Cores were processed in 5 cm depth intervals, excepting the top 50 cm, which were done at 1 cm depth intervals for a separate project. All sections were dried until no mass was lost between daily measurements and the soil bulk density was calculated from the mass of the dried sections and their initial wet volume. Percent organic matter was determined through loss on ignition (LOI) of ground samples. Percent organic carbon was calculated from LOI using a conversion equation for salt marsh soils developed by Craft et al. [3], from which the carbon density was determined for each sample by multiplying the bulk density by the organic carbon fraction. The carbon density of the 1 cm depth intervals was averaged into 5 cm subsections as displayed in the data.
