# Supplementary material for: High resolution carbon stock and soil data for three salt marshes along the northeastern coast of North America
Source: Data Brief. 2018 Jul 21;19:2438–41. doi: 10.1016/j.dib.2018.07.037 (PMC6141490; doi:10.1016/j.dib.2018.07.037)
Supplement: Supplementary file 1 — Transparency document [file mmc1.docx]

None of the authors have any financial, personal interest, or belief that would serve as a conflict of interest with this study or its publication.

All authors have agreed to this submission which is original work and has not received prior publication and is not under consideration for publication elsewhere. Data described and included constitute the raw data used to produce an associated research article in *Geoderma* [1].

[1] Van Ardenne LB, Jolicoeur S, Bérubé D, Burdick D, Chmura GL. 2018. The Importance of Geomorphic Context for Estimating the Carbon Stock of Salt Marshes. *Geoderma* 330: 264-275. https://doi.org/10.1016/j.geoderma.2018.06.003
